# Maxillary calcifying epithelial odontogenic tumor with sinus and buccal vestibule extension: a case report and immunohistochemical study

**DOI:** 10.1186/s13000-016-0582-3

**Published:** 2016-11-21

**Authors:** Cristina Munteanu, Daniel Pirici, Alex Emilian Stepan, Adrian Camen, Claudiu Margaritescu

**Affiliations:** 1 Department of Oral & Maxillofacial Surgery, University of Medicine and Pharmacy Craiova, Petru Rares 2, Craiova, 200349 Romania; 2 Department of Research Methodology, University of Medicine and Pharmacy Craiova, Petru Rares 2, Craiova, 200349 Romania; 3 Department of Pathology, University of Medicine and Pharmacy Craiova, Petru Rares 2, Craiova, 200349 Romania

**Keywords:** Calcifying epithelial odontogenic tumor, Immunohistochemistry, Maxillary sinus, Tumor aggressiveness

## Abstract

**Background:**

Calcifying epithelial odontogenic tumor (CEOT) is a rare benign neoplasia, locally aggressive, that tends to invade bone and adjacent soft tissues. This case report describes the thirteenth known case of CEOT with maxillary sinus extension and the second one that also involves the buccal vestibule mucosa with peculiar histopathological and immunohistochemical data.

**Case presentation:**

Here we report the case of a 45-year-old female with a CEOT diagnosed and treated at the Oral & Maxillofacial Surgery Department, County Clinical Emergency Hospital of Craiova, Romania. The clinical and imaging investigation revealed an intraosseous tumor developed from the left posterior maxilla with maxillary sinus and buccal vestibule mucosa extension. Histopathology found an epithelium-rich CEOT variant, but with scattered S100 positive clear cells, focal small rounded cementum-like deposits and areas with some degree of nuclear pleomorphism. The immunohistochemical investigations emphasised its local aggressiveness behavior with involvement of multiple molecular mechanisms that underlie tumor invasiveness. A subtotal maxillectomy was performed followed by defect reconstruction.

**Conclusions:**

We discuss the relevant clinicopathological features of an aggressive rare case of CEOT with maxillary sinus extension and buccal vestibule mucosa involvement. The immunohistochemical study suggests its utility in attempting to assess the degree of local tumor aggressiveness and thus in adopting the most efficient therapeutic attitude.

## Background

Calcifying epithelial odontogenic tumour (CEOT) is a rare benign epithelial odontogenic neoplasia that is characterized by the presence of amyloid-like material that may become calcified [1].

The first cases were reported by Thoma and Goldman [2] in 1946 as an “adenoid adamantoblastoma”, and in 1971 the term “calcifying epithelial odontogenic tumor” was generally accepted and adopted by the WHO [3].

It is one of the least frequent odontogenic tumors, its incidence ranging between 0.4 and 3% [4]. Although it is a benign tumor, sometimes can be locally aggressive infiltrating the surrounding jaw bones structures [5, 6], and even vital structures such as the brain [7]. Furthermore, in English literature it was reported a general recurrence rate ranging from 15 to 30%, mostly due to inadequate management [4, 8, 9]. On the other hand, malignant transformation appears to be a rare event, with less than 10 cases reported so far [10, 11].

We report here a case of intraosseous CEOT developed from the left posterior maxilla with maxillary sinus and buccal vestibule mucosa extension. A comprehensive immunohistochemical study was performed concerning the local aggressive behavior of this tumor. A written informed consent was obtained from the patient for research and publication purposes.

## Case presentation

### History

A 45 year old female presented to our institution with a diffuse swelling of the left maxilla which caused a mild tumefaction of the left cheek and obliteration of the buccal vestibule, left cheek pain during chewing with auricular and temporal irradiation, and masticatory disorders. The patient reported the onset of symptoms about 3 weeks before, but the exact duration of the swelling is not known. However, the patient remembered that about 3 years ago she was complaining of pain in the molar region of left maxilla and she addressed to her dentist. Without X-ray examination it was decided for extraction of the 2nd molar which presented palatal ectopia, but without caries and no abnormal mobility. Personal or family records were not significant, but noteworthy is that she worked about 10 years as an industrial dyer.

### Local physical examination

**Fig. 1:**
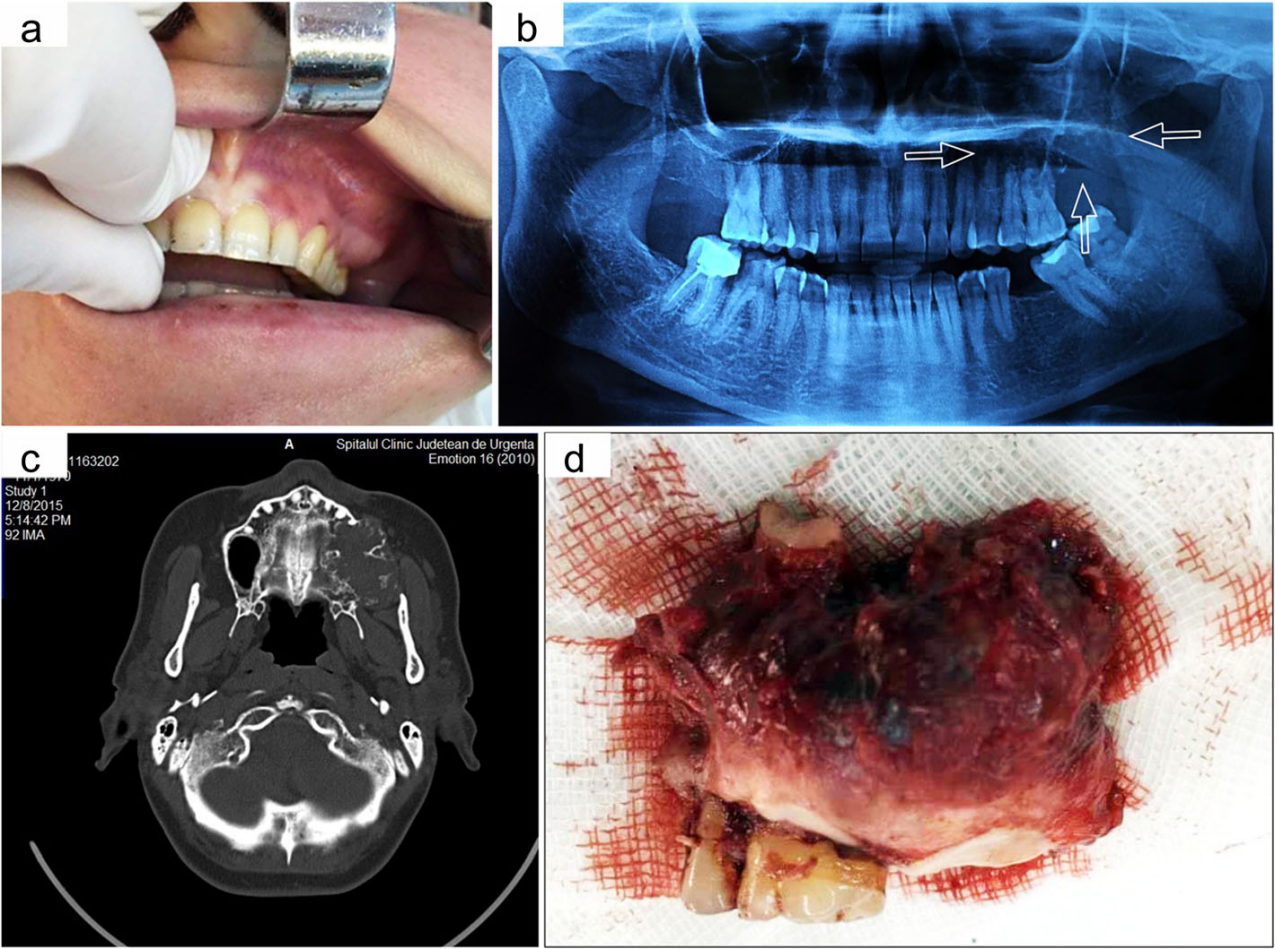
Clinico-radiological and gross pathological features of the tumor. **a** Intraoral view of the tumor with expansion on upper left buccal vestibule obliterations. **b** Orthopantomogram showing osteolytic image of left maxilla with few foci of calcifications. **c** Axial CT demonstrates radiolucency of the left maxilla that completely obliterated the maxillary sinus and involvement of the alveolar process with erosion of the cortical plate and invasion of the adjacent soft tissue. **d** Post-operative view of the lesion after surgical exposure. Gross features of the surgical specimens including the alveolar process with corresponding teeth

Extraoral examination revealed a mild diffuse swelling of the left cheek, most obvious at the posterior side of the zygomatic region. It was painless and hard in consistency on palpation. Intraoral examination revealed a diffuse swelling extending from the maxillary left first premolar to the tuberosity and retromolar regions that obliterated the buccal vestibule (Fig. 1a). The overlying mucosa was stretched but intact. On palpation, at the level of maxillary alveolar process the swelling was hard and painless but became fluctuating and slightly tender in the retromolar region.

### Radiographic and CT findings

Orthopantomogram showed a radiolucent lesion involving the left maxillary tuberosity, alveolar process, and the maxillary sinus with erosion of lateral wall and floor of the sinus (Fig. 1b). On computed tomography scan it was noticed an expansile, radiolucent lesion of the left maxilla (about 4 cm in diameter) with scattered areas of calcification which completely obliterated the left maxillary sinus (Fig. 1c). The scan showed tumor extension and involvement of the alveolar process (behind the first premolar with buccal cortical thinning, erosion and invasion of the soft tissue from retromolar region), maxillary sinus (with erosion of lateral and nasal walls, and floor of the sinus) and palatine process.

### Management

Based on history, clinical and imaging findings, a provisional diagnosis of benign locally aggressive odontogenic neoplasm of the left maxilla was considered. Under general anesthesia, a subtotal maxillectomy procedure was performed through Dieffenbach’s modification of Weber–Fergusons incision followed by defect reconstruction.

### Gross examination

The surgical specimen included almost the entire left maxilla, respectively the entire zygomatic process, the distal alveolar process with corresponding teeth, and limited area of the frontal and palatal processes, measuring 4.5 × 4 × 3 cm in total (Fig. 1d). The tumor eroded the floor and anterolateral wall of the maxillary sinus, occupying the entire left maxillary sinus being adherent to the lining mucosa but without its ulceration. Also, this tumor distorted the left alveolar process starting from the second bicuspid tooth up to the retromolar area, destroying the left maxillary tuberosity and distal to the third molar tooth becoming adherent to the vestibular mucosa but without its ulceration. The palatal process was partially involved but without palatal fibromucosa invasion. The tumor itself was relatively well defined, with smooth surface and firm in consistency. On cut section it appeared as solid, whitish gray with various amounts of calcification, and with sandy-like consistency.

### Histopathological examination

**Fig. 2:**
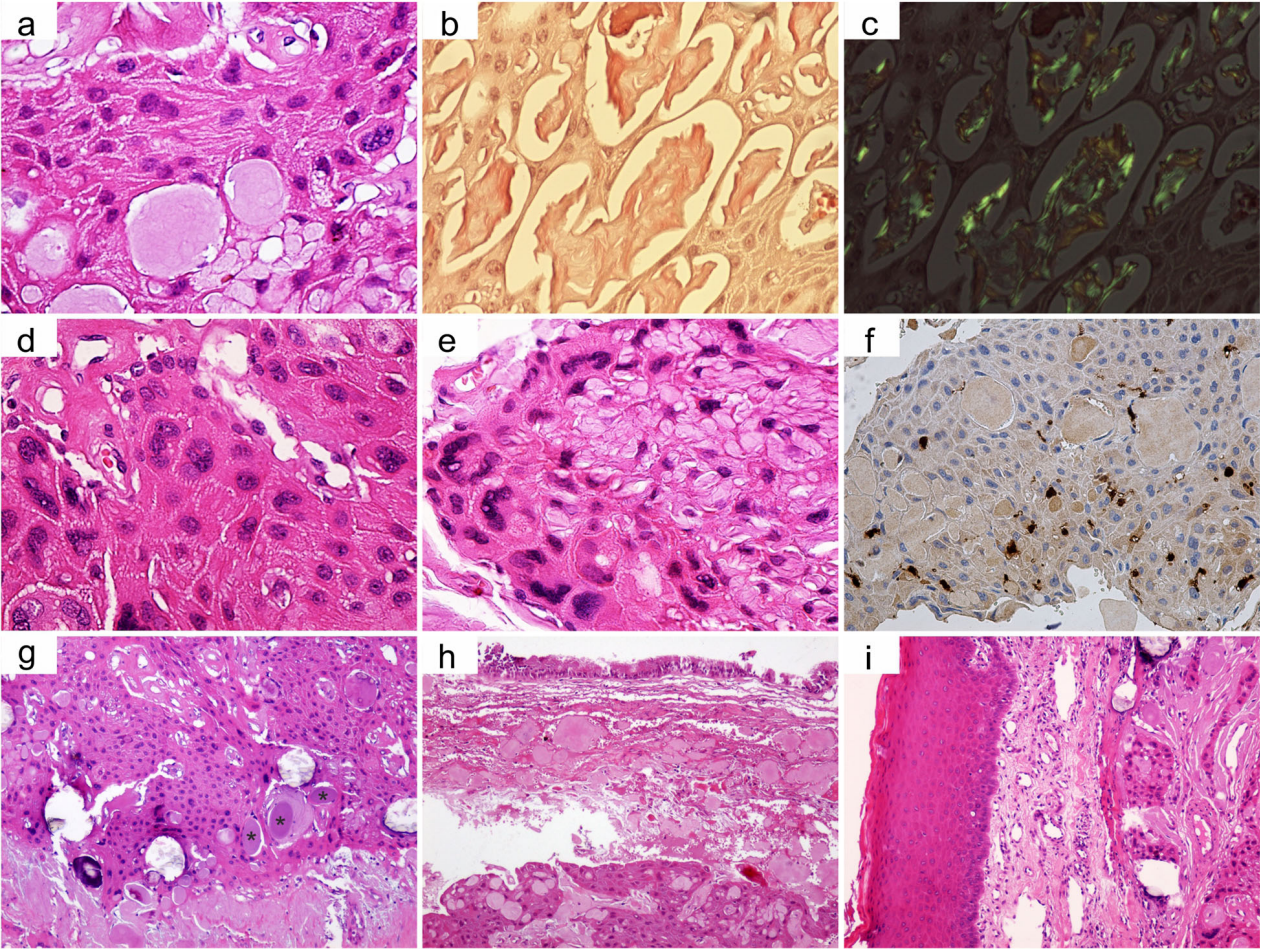
The main histomorphological features of the tumor. **a** The tumor was composed of polyhedral neoplastic cells with intercellular bridges and some degree of nuclear pleomorphism (hematoxylin and eosin [H&E]; original magnification × 40). **b** Homogeneous eosinophilic acellular material between neoplastic proliferations that stained positive for Congo red (Congo red stain; original magnification × 40). **c** Intercellular deposits with apple-green birefringence on polarized light examination of the Congo red stained sample (original magnification × 40). **d** Tumor areas with nuclear pleomorphism (H&E; original magnification × 40). **e** Tumor areas with nuclear pleomorphism and multinucleate giant cells (H&E; original magnification × 40). **f** Scattered S100 positive clear cells between polyhedral neoplastic cells (immunostaining, original magnification × 20). **g** Tumor comprises areas with cementum-like material (*) (H&E; original magnification × 10). **h** Tumor invasion of the maxillary sinus mucosa without involvement of the lining epithelium (H&E; original magnification × 10). **i** Tumor invasion of vestibular mucosa without involvement of the lining epithelium (H&E; original magnification × 10)

The tumor was composed of variable amounts of polyhedral eosinophilic epithelial cell islands, cords and strands in a fibrous stroma. These neoplastic cells had well-defined boundaries, intercellular bridges in focal areas and some degree of nuclear pleomorphism, including few bizarre nuclei (Fig. 2a). Between cells and within tumoral stroma, there was a conspicuous homogeneous eosinophilic acellular material that stained positive for Congo red (Fig. 2b). This material was confirmed as amyloid by examination in polarized light after Congo red staining, with the deposits exhibiting the characteristically apple-green birefringence (Fig. 2c). Some of the small round-shaped amyloid-like entities undergone calcification and only a few of them presented Liesegang rings. We also noticed the presence of areas with accentuated nuclear pleomorphism (Fig. 2d), some multinucleated giant cells (Fig. 2e), scattered S100 positive clear cells (Fig. 2f), and few clear cells with intracytoplasmic PAS-positive granules (glycogen). Altogether, the tumor did not raise the suspicion of another origin, as for example a primary or metastatic intraosseous squamous cell carcinoma. In some areas, between neoplastic proliferations were also observed small rounded cementum-like deposits (Fig. 2g). The tumor proliferations penetrated the cortical plates of maxillary bone invading the connective tissue of the maxillary sinus mucosa and the vestibular mucosa but without involvement of the lining epithelium (Fig. 2h, i).

### Immunohistochemical findings

The polyhedral neoplastic epithelial cells were diffusely cytoplasmic immunoreactive for CK 5/6 and CK19, while the clear cells were negative for these epithelial markers, exhibiting a nuclear and cytoplasmic positivity for the anti-S100 protein. Also the tumor polyhedral cells had an intense nuclear reactivity for p63, and with the cytoplasm slightly positive for vimentin, but negative for α-smooth muscle actin.

**Fig. 3:**
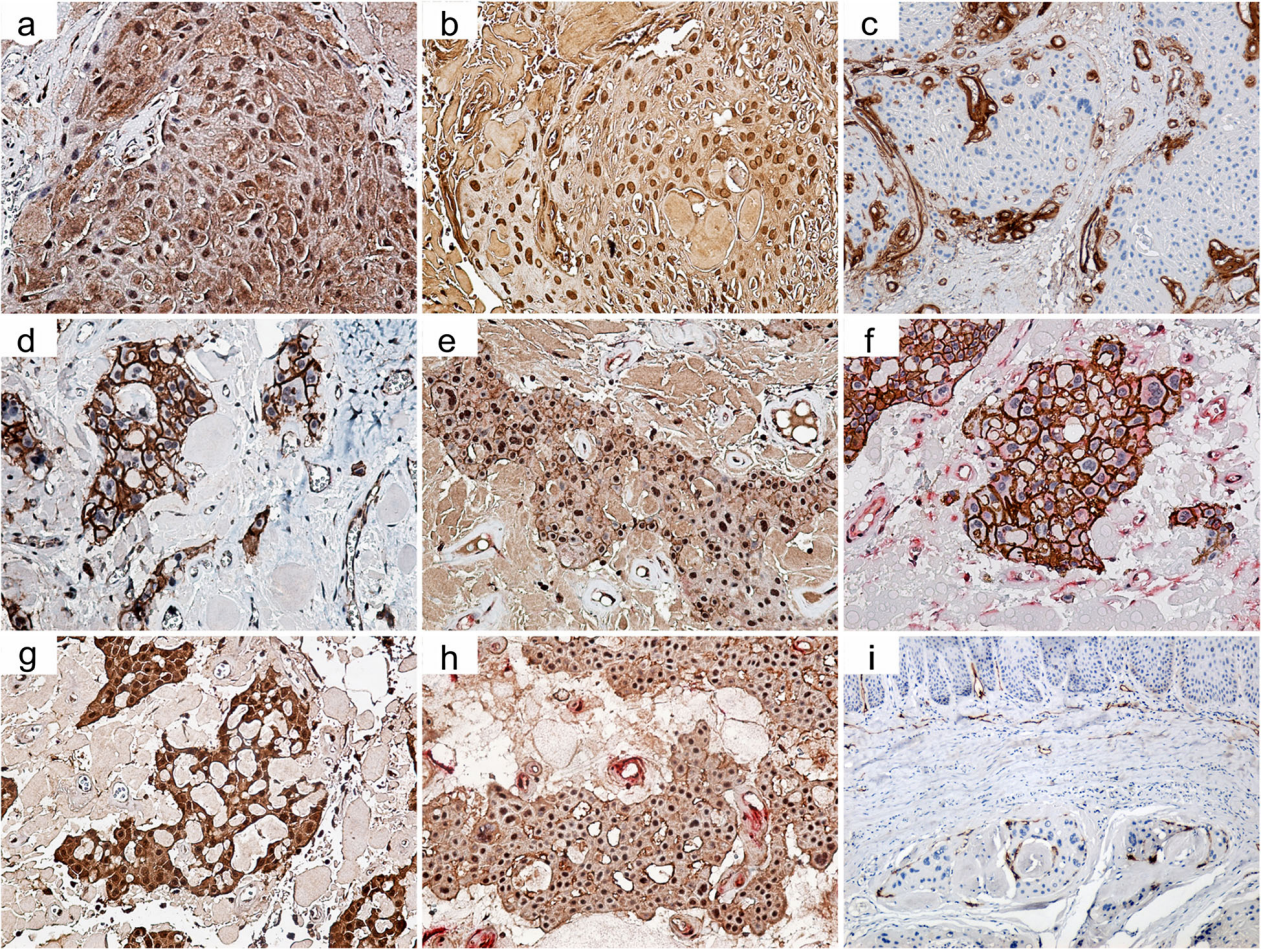
Immunohistochemical patterns of the tumor. **a**–**b** Neoplastic epithelial cells with strong nuclear and cytoplasmic staining for MMP-3 and MMP-9 (immunostaining, original magnification × 20). **c** Variable collagen IV reactivity around neoplastic epithelial proliferations (immunostaining, original magnification × 20). **d** Neoplastic epithelial cells from tumor advancing edge with strong membrane and cytoplasmic staining for Beta-catenin (immunostaining, original magnification × 20), **e** Neoplastic epithelial cells from tumor advancing edge with strong membrane and nuclear staining for N-cadherin (immunostaining, original magnification × 20). **f** Neoplastic epithelial cells from tumor advancing edge with E-cadherin (brown staining) and vimentin (red staining) cytoplasmic colocalization (double immunostaining, original magnification × 20). **g** Neoplastic epithelial cells from tumor advancing edge with strong cytoplasmic and nuclear reactivity for Slug 1 (immunostaining, original magnification × 20). **h** Neoplastic epithelial cells with strong membrane, cytoplasmic and nuclear reactivity for CXCR4 (double immunohistochemical detection CXCR4-brown staining and vimentin- red staining) (double immunostaining, original magnification × 20). **i** Podoplanin tumor cell reactivity, with a membranous pattern at the advancing edge, mainly at the periphery of tumor proliferations (immunostaining, original magnification × 10)

In order to explain its extremely aggressive behavior we investigated tumor cell immunoreactivity for a number of markers related to tumor invasiveness, as listed in Table 1. Thus, we observed expression of matrix metalloproteinases (MMP)-1, MMP-2, MMP-3, and MMP-9 mainly in the nuclei of tumor cells, while for MMP-3 and MMP-9 the immunoreactivity was also present in the cytoplasm of tumor cells and in the amyloid deposits (Fig. 3a, b). In addition, collagen IV reactivity was detected as a linear pattern around the tumor islands, sheets and nests but with variable thickness along the tumor tissue with discontinuities at the advancing edge (Fig. 3c).

**Table 1:** Characteristics of the antibodies utilised in the study

Antibody name	Clone	Company	Dilution	Staining pattern
MMP1	3B6	Santa Cruz Biotechnology, Inc. Dallas, USA	1:25	Nuclear
MMP2	2C1	Santa Cruz Biotechnology	1:25	Nuclear
MMP3	1B4	Santa Cruz Biotechnology	1:25	Nuclear, cytoplasmic, amyloid
MMP9	2C3	Santa Cruz Biotechnology	1:25	Nuclear, cytoplasmic, amyloid
E-cadherin	NCH-38	Dako, Glostrup Denmark	1:30	Membranous, cytoplasmic
N-cadherin	6G11	Dako	1:30	Membranous, nuclear
P- cadherin	H-105	Santa Cruz Biotechnology	1:30	Membranous, cytoplasmic, nuclear
Integrin β1	4B7R	Santa Cruz Biotechnology	1:30	Membranous (lost in some areas), cytoplasmic
Beta-catenin	β-catenin-1	Dako	1:100	Membranous, cytoplasmic
Vimentin	SP20	Thermo Fisher Scientific, Waltham, USA	1:200	Cytoplasmic
Twist 1	10E4E6	Leica Biosystems, Wetzlar, Germany	1:1000	Nuclear
Slug 1	1A6	Novus Biologicals, Abingdon, UK	1:50	Nuclear, cytoplasmic
CXCR4	polyclonal	Thermo Scientific	1:500	Membranous, cytoplasmic, nuclear
Podoplanin	D2-40	Dako	1:50	Membranous
Collagen IV	CIV 22	Dako	1:50	Basement membranes
Ki-67	MIB-1	Dako	1:50	Nuclear
Cytokeratin 5/6	D5/16 B4	Dako	1:50	Cytoplasmic
Cytokeratin19	b170	Leica Biosystems	1:150	Cytoplasmic
P63	7JUL	Leica Biosystems	1:25	Nuclear
S100	polyclonal	Dako	1:500	Cytoplasmic, nuclear
Actin (Smooth Muscle	1A4	Dako	1:40	Cytoplasmic
Actin, Smooth Muscle	polyclonal	Thermo Scientific	1:100	Cytoplasmic

Investigation of markers involved in cell adhesion showed a positive reaction with prevailing membranous pattern in the tumor cells for E-, N- and P-cadherin, Integrin β1 and Beta-catenin. However, as a peculiarity of the tumor advancing edge, where small groups or cords of infiltrating tumor cells were present, the staining pattern became also cytoplasmic for all cadherins, Integrin β1 and Beta-catenin (Fig. 3d), and even nuclear for N- (Fig. 3e) and P-cadherin. In these areas the membranous immunoreactivity decreased and at least for E-cadherin we noticed an increased cytoplasmic colocalization with vimentin (Fig. 3f).

Investigation of other markers involved in the epithelial-mesenchymal transition process revealed mostly a nuclear reactivity of tumor cells for Twist 1 and Slug 1 (Fig. 3g) that seemed to be more intense at the advancing edge. Also, the chemokine receptor −4 (CXCR4) reactivity was also noticed in tumor cells with membranous, cytoplasmic and even nuclear pattern (Fig. 3h) Moreover, the podoplanin tumor cell reactivity, with a membranous pattern, was more obvious at the advancing edge, at the periphery of tumor proliferations (Fig. 3i). However the Ki-67 labeling index in tumor cells was less than 3% and without any significant difference regarding the tumor topography.

A final diagnosis of intraosseous CEOT with maxillary sinus and buccal vestibule submucosa extension was made on the basis of the above findings. After 12 months of follow-up, the patient did not present any clinical or radiographic evidences of recurrence.

## Discussion

CEOT is a rare benign odontogenic neoplasm of epithelial origin with locally aggressive behavior that so far does not exceed 200 reported cases [10]. In our experience this is the second case of CEOT from a total of 231 cases of odontogenic tumours diagnosed in our hospital since 1990. The general epidemiological profile reported in the literature for this odontogenic tumor shows a mean age at presentation of 40 years, with no gender predilection and mandible as the most commonly affected jaw bone [1]. Similarly to other authors [4], our results indicated the intermediary layer of the enamel organ as the most probable origin for this type of odontogenic tumor.

One of the peculiarities of the presented case is its intraosseous development in the left posterior maxilla with extraosseous involvement of maxillary sinus and buccal vestibule mucosa. Reviewing the literature, we found other 12 cases of CEOT with an extension to the maxillary sinus, which are summarized in Table 2. Thus, the present case is the thirteenth CEOT with maxillary sinus extension and the second one that also involved the buccal vestibule mucosa. These aggressive CEOT tumors seem to develop at a median age of 35 years with no gender predilection, in association or not with impacted teeth and no predilection for the right or left maxilla [4, 12–22]. Histopatologically they had the same features as a conventional COET, only one case being reported with the clear cell variant and another one with the cystic variant. A particular feature of the present case was the presence of scattered S100+ clear cells and deposits of cementum-like material. In more than half of these cases, CEOT evolved extraosseous with extension in the adjacent soft tissues, and to the neighboring natural cavities (oral cavity, orbit, nasal fossae, and ethmoidal air cells) [4, 12–22].

**Table 2:** Clinicopathological features of currently reported cases of CEOT involving the maxillary sinus

Reference	Age	Gender	Teeth involvement	Maxillary bone involved	Extraosseous extension besides maxillary sinus	Microscopic features	Follow up
Gon, 1965 [14]	35	Female	First molar	Right maxilla	No	Conventional	NP
Stimson et al., 1968 [19]	35	Male	Third molar	Left maxilla	Left nasal fossa	NF	NF
Lee et al., 1992 [16]	27	Female	Second premolar	Left maxilla	No	Conventional	7 years.
Bridle et al., 2006 [12]	30	Female	Displacement of the second premolar and first molar	Right maxilla	Displacement of the eye globe	Conventional	NP
Gopalakrishnan et al., 2006 [15]	15	Male	Second molar	Left maxilla	Nasal cavity, narrowing and compressing the inferior meatus	Cystic variant	1 year
Mohtasham et al., 2008 [17]	18	Male	No	Right maxilla	None	Conventional	NP
da Rosa et al., 2011 [13]	33	Female	No	Left maxilla	Lateral wall of nasal cavity, soft tissue, masseter, orbicularis oris, medial pterygoid	Conventional	NP
Sahni et al., 2012 [4]	52	Male	No	Right maxilla	No	Conventional and clear cell	3 years
Müller et al., 2012 [18]	36	Male	No	Right maxilla	No	Conventional	4 years
Carrero et al., 2014 [20]	69	Male	Third molar	Left maxilla	No	Conventional	8 years
Foroughi et al., 2015 [21]	34	Female	No	Recurrence in left maxilla	Orbit and ethmoidal air cells superiorly and the nasal airway medially	Conventional	8 years
Rani et al., 2016 [22]	48	Female	No	Right maxilla	Connective tissue of oral mucosal	Conventional	1 year
Present case	45	Female	Palatal ectopia of the second molar	Left maxilla	Buccal vestibule mucosa	Conventional with few clear cells and cementum-like components	0.6 years

*NP* Not Performed, *NF* Not Found

It is well know that this odontogenic tumor can be locally aggressive, and exhibit up to 15% recurrence rates, especially in cases treated by conservative techniques [9].

Throughout time several prognostic factor have been proposed to predict the behavior of such tumors and to estimate their recurrence risk. Thereby, clinical features such as size, anatomic site, and health status of the patient have been shown to influence prognosis of these tumors [4, 12]. It is widely accepted that maxillary CEOTs tend to be more aggressive and involve much easier the surrounding vital structures than mandibular tumors. As it is also mentioned in the literature, and as well as in our case, amyloid deposits were not very abundant, with calcifications being even less present, a fact that might suggest a more aggressive behavior [23].

Also, CEOTs with clear cells seem to be more aggressive, with a higher rate of recurrence (22%) and frequently associated with cortical bones perforation [24]. In the present case we showed that few S100+ cells were scattered between eosinophilic polygonal neoplastic cells. This reactivity reflects the dendritic nature of these cells and their clear cell morphology could be an effect of tumor microenvironment on the Langerhans cells migrating in the tumor as a result of the host’s immune response [25]. Such morphological changes could represent a mechanism by which tumours can escape immune surveillance and gain a more aggressive biologic behavior.

For a better understanding of the molecular mechanisms that underlie the aggressiveness of this odontogenic tumor we planned to investigate the expression patterns of a number of markers known to be involved in tumor invasiveness.

First we have checked the tumor cells reactivity for the main MMPs (MMP-1, −2, −3, −4) known to be involved in extracellular matrix remodeling. Thus we noticed a tumor nuclear reactivity for all MMPs but with high intensity especially for MMP-3 and MMP-9, which were also expressed in the cytoplasm and within amyloid deposits. Henriques et al. [26] found a higher MMP9 expression both in tumor cells and stroma, for keratocystic odontogenic tumors and ameloblastomas, that might justify their locally aggressive behavior. The nuclear tumor cell expression for the all 4 investigated MMPs reported by us might also be linked to new functional roles, such as activation of some cancer-related signaling pathways that could promote tumor proliferation, and most likely invasion and metastasis [27]. Moreover, studying the collagen IV expression, we noticed absence or discontinuity in reactivity at the invasion front, a fact that could justify the local aggressiveness of the presented case. Other studies pointed out that collagen IV expression could be correlated with the growth and aggressiveness of odontogenic tumors [26, 28]. Thus MMP-9 and collagen IV could be useful markers in assessing the degree of local aggressiveness of these odontogenic tumors, including the CEOTs.

Investigating the epithelial–mesenchymal transition (EMT) process, we have found that tumor cells were reactive for all investigated cadherins, for Integrin β1 and Beta-catenin with a gradual transition from the membranous pattern to a cytoplasmic pattern toward the advancing edge. In addition, we have observed even a nuclear reactivity for N- and P-cadherin. Moreover, as a sign of active EMT, we found an increased cytoplasmic reactivity for vimentin and a prevailing nuclear Twist 1 and Slug 1 expression in tumor cells, especially at the tumor’s advancing edge. Our results were confirmed in the literature by cases of ameloblastomas, where it was also been noticed an E-cadherin downregulation simultaneously with an upregulation of podoplanin, β-catenin, and CD44v6 at the tumor advancing edges, suggesting their involvement in mediating collective cell migration and local invasiveness [29]. Moreover, it has been reported that transcription factors such as Snail, Slug, SIP1, and Twist could have differential roles in mediating local invasiveness in ameloblastomas [30]. Also, in the present study we have observed a podoplanin expression at the advancing edge, mainly at the periphery of tumor proliferations, suggesting its involvement in tumor invasiveness. In fact, it is well known that this transmembrane sialomucin is involved in odontogenesis and in promoting local invasion of various odontogenic tumors [29].

A series of studies reported the role of CXCR4 in modulation of cancer cells migration in several human malignancies, especially due to increase production of MMPs [31, 32]. Here we have observed membrane and nuclear CXCR4 reactivity in CEOT cells regardless of tumor topography (tumor center versus the advancing edge), suggesting the involvement of this chemokine in mediating locally tumor cell invasion or even a potential malignant evolution of this tumor.

However, despite its aggressive behavior, we found that less than 3% of tumor cells were positive to Ki-67, a value that falls within the range (0.27–10%) reported by the literature for this kind of tumors [33]. Higher values were recorded in CEOTs with malignant transformation, with at least three-fold increase in the suspected malignant area compared to benign areas [11]. A reduced level of Ki-67 index together with the absence of any clinical finding of metastasis, with no vascular invasion or atypical mitotic figures, justifies the benign character of the tumor in the current case.

Summarizing our results we can conclude that the peculiar locally aggressive behavior of the presented case is the result of the involvement of multiple molecular mechanisms. Thus investigating such biomarkers could be useful in assessing the degree of local aggressiveness and even the malignant potential of these odontogenic tumors, suggesting also the most efficient type of therapeutical approach.

## Conclusions

This case report describes the thirteenth known case of CEOT with maxillary sinus extension and the second one that also involves the buccal vestibule mucosa. Histopathologically, this case corresponds to an epithelium-rich CEOT variant, but with scattered S100+ clear cells and deposits of cementum-like material. The immunohistochemical investigation confirms its aggressive behavior by tumor overexpression of biomarkers involved especially in extracellular matrix degradation, cell adhesion and the EMT processes, and even a potential malignant evolution of this tumor. However, our case did not fulfill the other criteria of malignancy thus was deemed a benign tumor. A radical surgery resection with defect reconstruction was performed and after 12 months following the treatment there was no signs of recurrence.

## Abbreviations

CEOT: Calcifying epithelial odontogenic tumor
CK: Cytokeratin
CT: Computed tomography
CXCR-4: Chemokine receptor type 4
EMT: Epithelial to mesenchymal transition
H & E: Hematoxylin and eosin
MMP: Matrix metalloproteinase
PAS: Periodic acid–Schiff
